# Potential Role of Cannabinoid Type 2 Receptors in Neuropsychiatric and Neurodegenerative Disorders

**DOI:** 10.3389/fpsyt.2022.828895

**Published:** 2022-06-14

**Authors:** Hiroki Ishiguro, Berhanu Geresu Kibret, Yasue Horiuchi, Emmanuel S. Onaivi

**Affiliations:** ^1^Department of Clinical Genetics, Graduate School of Medical Science, University of Yamanashi, Kofu, Japan; ^2^Department of Neuropsychiatry, Graduate School of Medical Science, University of Yamanashi, Kofu, Japan; ^3^Department of Biology, College of Science and Health, William Paterson University, Wayne, NJ, United States; ^4^Department of Psychiatry and Behavioral Sciences, Tokyo Metropolitan Institute of Medical Science, Tokyo, Japan

**Keywords:** cannabinoid CB2 receptors, neuropsychiatric disorders, neurodegenerative (ND) disorders, neuronal and glia activation

## Abstract

The endocannabinoid system (ECS) is composed of the two canonical receptor subtypes; type-1 cannabinoid (CB1R) and type 2 receptor (CB2R), endocannabinoids (eCBs) and enzymes responsible for the synthesis and degradation of eCBs. Recently, with the identification of additional lipid mediators, enzymes and receptors, the expanded ECS called the endocannabinoidome (eCBome) has been identified and recognized. Activation of CB1R is associated with a plethora of physiological effects and some central nervous system (CNS) side effects, whereas, CB2R activation is devoid of such effects and hence CB2Rs might be utilized as potential new targets for the treatment of different disorders including neuropsychiatric disorders. Previous studies suggested that CB2Rs were absent in the brain and they were considered as peripheral receptors, however, recent studies confirmed the presence of CB2Rs in different brain regions. Several studies have now focused on the characterization of its physiological and pathological roles. Studies done on the role of CB2Rs as a therapeutic target for treating different disorders revealed important putative role of CB2R in neuropsychiatric disorders that requires further clinical validation. Here we provide current insights and knowledge on the potential role of targeting CB2Rs in neuropsychiatric and neurodegenerative disorders. Its non-psychoactive effect makes the CB2R a potential target for treating CNS disorders; however, a better understanding of the fundamental pharmacology of CB2R activation is essential for the design of novel therapeutic strategies.

## Introduction

This is an important update on the molecular framework for the therapeutic potential of targeting CB2 cannabinoid receptor ligands, based on reviews, and previous work by our group (1–3) and others. There has been an explosion of research, and new knowledge on the emerging and evolving endocannabinoid system (ECS) to an expanded endocannabinoidome (eCBome) to include other putative cannabinoid receptors (CBRs), lipid molecules, and previously unknown targets of endocannabinoids (eCBs). Delta-9-tetrahydrocannabinol (Δ^9^-THC) is the most abundant psychoactive, and most studied cannabinoid among the over 500 different compounds present in the cannabis plant (4). The expanding ECS which includes different protein and lipid mediators is involved in the pathophysiology of neuropsychiatric disorders and targeting this system might be of benefit in the management of a number of CNS diseases (5).

CB1Rs and CB2Rs are G-protein coupled receptors (GPCRs) (6). It turned out that CB1Rs are the most abundant GPCRs in the brain, and are expressed in motor function, reward and cognition (7) whereas CB2Rs was reported to be predominantly found in peripheral tissues of immune origin, (8–10). Our previous understanding was that CB2Rs were not expressed in the brain (10–12), but now there are numerous *in vitro* and *in vivo* studies showing neuronal and glial expression of CB2R in many parts of the CNS (1, 13). The expression of CB2Rs in dopamine neurons have been documented (14). Unlike CB1Rs, very little is known about the relevance of functional neuronal CB2Rs as they were previously considered to be absent from the brain, hence the role of CB1Rs have been more established and well-studied in comparison to the CNS effects of CB2Rs. However, as others and we have demonstrated at least in preclinical studies CB2Rs are post-synaptically expressed and just like CB1Rs are expressed in some pre-synaptic terminals (15, 16). The low expression of CB2Rs is now recognized but their expression is increased during injury and inflammation, with their upregulation during CNS disorders is providing a basis and focus of attention for the use of CB2R ligands in the neuroprotective and anti-inflammatory activity associated with neuropsychiatric and neurodegenerative disorders. With the increasing research and use of marijuana, some cannabinoids that lack CNS psychoactive effects have been associated with a role CB2Rs as we reviewed (16, 17). Furthermore, there is increasing association of inflammation and neuro-immune crosstalk in neuroinflammatory and neurodegenerative disorders. This review provides an update (1–3) and our pioneering basic research and those of others on the increasing putative neuro-immuno-ECS-CB2Rs in neuropsychiatric and neurodegenerative disorders using a narrative review methodology from currently used search engines. Therefore, this updated review (see also 1–3), discusses recent advances in the integrated neuro-immuno-cannabinoid network, and summarizes the potential role of CB2Rs in neuropsychiatric and neurodegenerative disorders. There are challenges with a paucity of research on the interaction and contribution of both CB1Rs and CB2Rs both in the periphery and CNS disorders. However, the determination of the crystal structures of CB1R and CB2R that revealed functional profile and relationship of CB2R antagonism versus CB1R agonism, provides insights for developing allosteric modulator and dual steric ligands targeting both receptors.

### Endocannabinoidome: Insights Into the Expanding Endocannabinoid System With a Focus on Cannabinoid Type 2 Receptors

**FIGURE 1:**
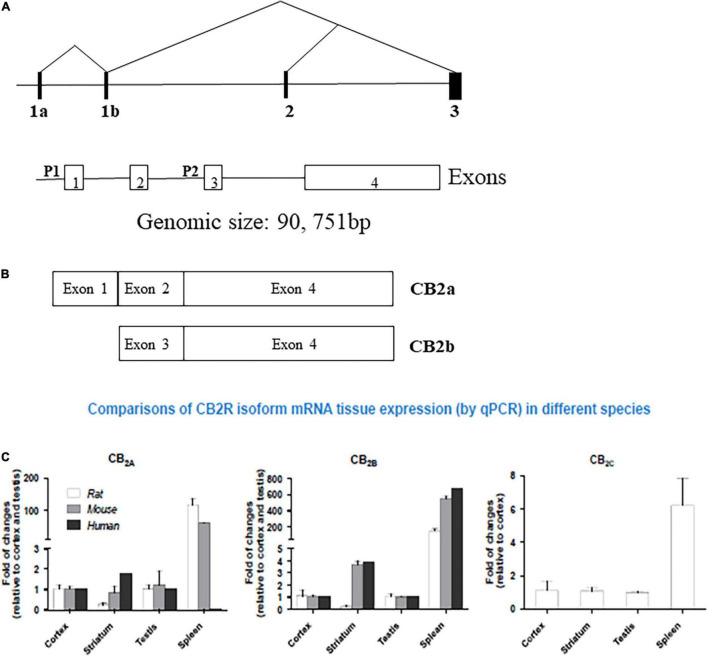
Human hCB2 genomic structure and isoforms: **(A)** human *CNR2* genomic structure. P represent promoters and exons are open boxes with the exon numbers. **(B)** CB2a and CB2b isoforms and **(C)** comparison of CB2R isoform mRNA in Human, Rat, and Mouse tissue expression by qPCR. (See also reference Liu et al. (38)).

The expanding ECS include CB1Rs, CB2Rs, and other putative cannabinoid receptors (CBRs), like GPR-55, TRPV1, PPAR and their lipid molecules, and previously unknown targets of endocannabinoids (eCBs) which are endogenous substances that bind to CBRs to mediate their physiology actions (18, 7). 2-AG and AEA are well-characterized eCBs (19) with reports of opposing actions by differential activation of many signaling pathways that are associated with many biological systems. Other lipid molecules including peptide eCBs, fatty acid amides, ethanolamides, and monoacylglycerol molecules with CBRs provides the platform from the ECS to eCBome. It turned out that the most abundant G-protein coupled receptors (GPCRs) in the human brain is for CB1Rs that has been well characterized compared to the accumulating knowledge on CB2Rs that also belong to this family of GPCRs. While the tissue distribution of both CB1Rs and CB2Rs are mostly different, some overlap exists in brain regions where they may work together or independently using similar or different signaling cascades (3, 6, 20–22) resulting in similar or opposing effects (17). There are abundant distribution of CB1Rs in pre-synaptic terminals not only in brain areas associated with the control of emotionality and motor function, but also in other brain regions. In contrast to the pre-dominantly presynaptic localization of CB1Rs, CB2Rs are mostly in post-synaptic terminals. However, just like CB1Rs that are also expressed in some post-synaptic terminals, CB2Rs are also expressed in some pre-synaptic terminals (1, 2, 4). For example, in the rat brain, using *in vitro* preparations, there is the presence of postsynaptic intracellular CB1Rs and CB2Rs following activation of TRPC4/5-like channels, and a demonstration of the presence of functional presynaptic CB2Rs inhibiting [^3^H]-glutamate release at subthalamo-nigral neuron terminals that are involved in motor control (23). Although CB1Rs and CB2Rs are both GPCRs with 44% similar homology and molecular structure, but with known different cellular expression and distribution patterns with functional specificity (Figure 1) (24). First cloned in 1993, CB2R is located on human chromosome 1p36 (8, 25–28) and mouse chromosome 4. Earlier studies could not find CB2Rs in the brain tissue and suggested that CB2Rs were absent in the brain and they were considered as peripheral CBRs, since *in situ* hybridization (ISH) and Northern blot analysis failed to detect CB2R mRNA in rat, mouse, and human brains (8, 9, 29, 30). However, studies conducted in our laboratory and by others identified the presence of CB2Rs throughout the CNS using different methods including ISH, immunostaining and gene expression (17, 31–38). Emerging evidence also shows that significant CB2R mRNA in the mouse cerebellum (39, 40), cortex (38, 41–43), striatum (9, 41), hippocampus (43), amygdala (16, 41), brainstem (1), and retina (44), and in the globus pallidus of non-human primates (43). In addition, some previous studies reported the discovery and functional characterization of CB2Rs in neural progenitor cells, neurons, glial and endothelial cells (13, 35, 45–47). Furthermore, two CB2R isoforms, CB2A and CB2B, have been characterized in the rodent and human brain, with CB2A, CB2B and CB2C only in the rat brain (Figure 1C) (38) along with a new CB2 transcript that has been found in mouse and monkey B lymphocytes (48). The evidence clearly suggests the expression of CB2R in the brain in addition to their expression in the periphery. With two separate promoters and 4 exons *CNR2* gene is almost four times as large as *CNR1* in genomic size, with CB2a and CB2b specie specific isoforms for brain and immune differential tissue expression profile, respectively (49) (Figure 1).

**FIGURE 2:**
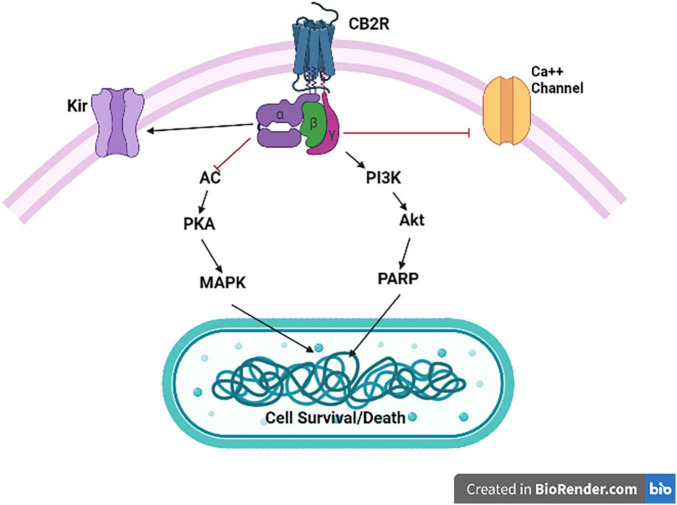
Cannabinoid type 2 receptor signaling. CB2R activation is associated with Gαi/o-dependent inhibition of AC activity and Gβγ-dependent activation of the different MAPK cascades. CB2R activation also results in inhibition of specific calcium channels (Ca^++^), and enhance opening of inwardly rectifying potassium (K_ir_) channels.

The activation of CB2Rs inhibits adenylyl cyclase (AC) activity and initiates MAPK and phosphoinositide 3-kinase (PI3K)-Akt pathways (Figure 2) with subsequent activation of the Jun N-terminal protein kinase (JNK), extracellular signal-regulated kinase (ERK)1/2 and p38 (50, 51). In addition, CB2R agonists results in increased synthesis of ceramide, a sphingolipid messenger, particularly in tumor cell lines, which induces apoptotic cell death (52). CB2Rs are mainly expressed post-synaptically and their activation inhibits postsynaptic neuronal function through membrane potential hyperpolarization (53), and also reported presynaptically in some terminals. Thus, CB2Rs are involved in modulating a variety of behavioral effects in the CNS with reports that CB2Rs modulate food intake, body weight (54, 55), depression and anxiety (14, 56), drug addiction (57, 58) and schizophrenia-like behavior (59). Brain CB2Rs are expressed at low levels under physiological conditions; however, in pathological conditions, such as neuropathic pain (60), stroke (61), traumatic brain injury (TBI) (62), neurodegenerative diseases (57, 63, 64) or drug addiction (65, 66), their expression is enhanced and up-regulated. The inducible nature of CB2Rs during events with underlying inflammation can make them a potential therapeutic target and ligands that activate or inhibit the activity of CB2Rs might be used to treat different disorders without causing profound adverse drug and intoxicating effects (67). As our understanding of the involvement of the ECS in different CNS physiologic and pathologic conditions increases, the ECS has gained a special attention as a potential therapeutic target and in designing safe and effective drugs to treat CNS disorders (68). Furthermore, understanding the underlining mechanisms for the involvement of the ECS in different pathological conditions including neuropsychiatric disorders is important in early diagnosis and treatment of these disorders. The expanded ECS, the (eCBome), that has been identified include *N*-acylethanolamines (NAEs), 2-acylglycerols (2-AcGs), *N*-acyl-amino acids, *N*-acyl-dopamine and *N*-acyl-serotonins (5). As studies unravel the complexity of this system, the interest in utilizing the eCBome system as a potential therapeutic target for the treatment of neuropsychiatric disorders is increasing (24, 5). In addition, previous studies revealed that targeting the CB1R is associated with adverse effects including anxiety, depression and even suicidal ideation (69, 70), and hence the search for a new therapeutic targets with minimal adverse effect is gaining special attention. Recent studies showed that targeting the CB2Rs, unlike the CB1Rs, is safe and effective and that CB2Rs might be new possibilities for safe targeting of the eCBome system (71). Therefore, there is increased research focus on the effects of neuronal function and behaviors induced by CB2R modulation and potential therapeutic applications (3–24). CB2Rs and their variants share an immunological basis that has been associated with endophenotypes of reduced immune function. Specifically, CB2R neuro-immune crosstalk may underlie chronic inflammation that is commonly observed in neuropsychiatric and neurodegenerative disorders. The association of CB2Rs with neuron-microglia crosstalk as target for reduction of the neuroinflammatory process could be considered as a target for the reduction of M1 action and induction of M2 activation. Thus, the molecular features, role and expression of CB2Rs in different neuronal and microglial phenotypes (MO, M1, and M2) will provide structural frame work in designing CB2R ligands in neurological disorders associated with neuro-inflammation. Of note, we have demonstrated and reported the expression of CB2Rs in neuronal, glial, and endothelial cells in the CNS and its function that is associated with neuropsychiatric and neurological disorders with neuroinflammation (17).

## Cannabinoid Type 2 Receptors in Neuropsychiatric and Neurodegenerative Disorders

**FIGURE 3:**
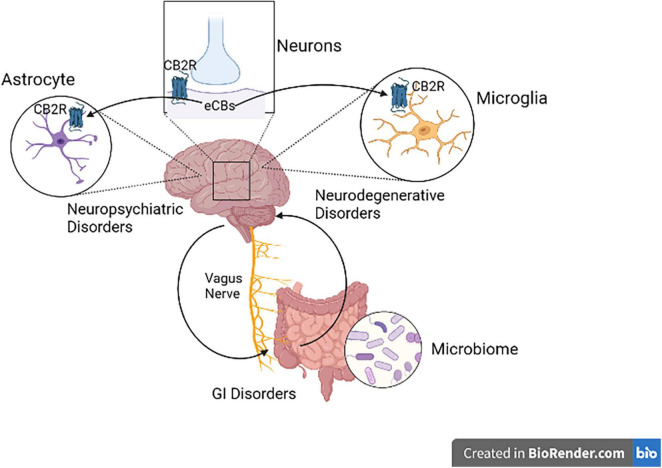
Cannabinoid type 2 receptor neuro-immune crosstalk in neuropsychiatric disorders. Activation of postsynaptic neuronal CB2Rs by cannabinoids alters neuronal network and function. CB2R might be a potential therapeutic targets in neuropsychiatry, since CB2R activation switches microglia to an anti-inflammatory state during injury. CB2Rs found in astrocytes also play a great role in Astrocyte-Neuron-Glia signaling.

Research in animal models using different molecular techniques like immunohistochemistry, Western blotting, gene expression and a battery of behavioral tests have identified the involvement of elements of the ECS particularly CB2Rs (Figure 3) in models of CNS function and dysfunction. Previous studies reported evidence for the involvement of CB2Rs in neuropsychiatry (34–37, 45, 72, 73). The expression of CB2Rs in healthy nervous system is low but inducible with varying perturbation that lead to enhanced and up-regulation associated with neurodegenerative and neuropsychiatric disorders. New lines of evidence now indicate that CB2R upregulation could serve as a neuroprotective mechanisms associated with a number of physical or mental injury. As the clinical and functional implications of neuronal CB2Rs in the brain is gradually becoming clearer, more research is unraveling their contribution in neuropsychiatry and global drug addiction (29). Currently there are no effective therapy and cure for most of neuropsychiatric disorders. Besides, the available conventional therapies are associated with a wide range of unwanted adverse drug effects. Hence there is a need for the search of new therapeutic targets for the development of safe and effective medications for preventing or retarding the disease process in neuropsychiatric disorders (74). Studies reviewed in the previous sections demonstrated the role of CB2Rs in the regulation of physiologic functions and hence they can be utilized as a potential target in neuroinflammatory and neurodegenerative disorders (75, 76), but requires further clinical trials. Furthermore, dissociating CNS psychoactivity and adverse side effects from therapeutic effects is a desirable goal. Below we briefly review evidence supporting the putative role of CB2Rs in various neuropsychiatric and neurodegenerative conditions. The preclinical and clinical role of CB2Rs in these disorders are summarized in Tables 1–4.

**TABLE 1:** Pre-clinical data on the role of CB2Rs in neuropsychiatric disorders.

Disorder	Model/paradigm	CB2R manipulation	Dose used	Outcome	References
Anxiety-like behavior	Chronic mild stress/ Elevated plus maze	JWH015	1–20 mg/kg	Induced angiogenesis	(34, 35)
	Marble burying	GW405833	0.3–100 mg/kg	Induced anxiolysis	(81)
	Chronic mild stress/zero maze	JWH015	20 mg/kg	Reduced anxiety like behavior	(82)
		AM630	3 mg/kg	Increased anxiety-like behavior	
	Light-dark box, elevated plus maze	AM630	1–3 mg/kg	Induced anxiogenesis and anxiolysis after acute and chronic administrations	(56)
		CB2xP mice		No response to anxiogenic-like stimuli	
	Light-dark box, elevated plus maze	*Cnr2–/–* mice		Enhanced anxiety-like behavior	(59)
	Light-dark box, elevated plus maze, forced swim and tail suspension	DAT-*Cnr2* cKO mice		Increased anxiety-like behavior	(14)
		JWH133	5 mg/kg		
Depression	Tail suspension, novelty-suppressed feeding test, chronic mild stress	CB2xP mice		Reduced immobility time	(80)
	Light-dark box, elevated plus maze	*Cnr2–/–* mice		Developed depressive-like behavior	(59)
	Forced swim and tail suspension	DAT-*Cnr2* cKO mice		Increased immobility time	(14)
		JWH133	5 mg/kg		
	Forced swim test	GW405833	30 mg/kg	No change in time spent immobile	(85)
	Chronic mild stress	JWH015	20 mg/kg	Enhanced CB2R protein level	(36, 45)
	Chronic mild stress	CB2xP mice		Reduced CB2R mRNA	(80)
Schizophrenia	Early maternal deprivation in rats			Increased CB2R immunoreactivity in the hippocampus	(95)
	MK-801, methamphetamine	AM630	3 and 30 mg/kg	Didn’t affect pre-pulse inhibition alone but enhanced MK-801 or methamphetamine induced effect	(98)
	MK-801	JWH015	1, 3, and 10 mg/kg	Enhanced pre-pulse impairment caused by MK-801	(99)
	Acoustic pre-pulse inhibition	*Cnr2–/–* mice		Decreased pre-pulse inhibition	(59, 100)
	MK-801, pre-pulse inhibition	*Cnr2–/–* mice		AM630 inhibited the ability of VU0467154 to reverse disruption of pre-pulse inhibition	(101)
		AM630	10 mg/kg		
Addiction	Self-administration, conditioned place preference	CB2xP		Decreased cocaine motor sensitization and self-administration	(109)
	Open field test	*Cnr2–/–* mice		Enhanced cocaine motor sensitization	(59)
	Open field, conditioned place preference	DAT-*Cnr2* cKO mice		Increased psychostimulant induced motor sensitization and conditioned place preference	(58)
		JWH133	3 mg/kg	JWH133 inhibited cocaine and nicotine induced conditioned place preference	
	Open field, conditioned place preference	*Cnr2–/–* mice		JWH133 blocked cocaine locomotion and self-administration	(33, 110)
		JWH133	10 and 20 mg/kg		
	Drug self-administration under fixed and progressive ration	β-caryophyllene		Attenuated methamphetamine self-administration	(112)
		*Cnr2–/–* mice		Blockage of β-caryophyllene induced reduction in methamphetamine self-administration	
	Alcohol consumption and place preference	*Cnr2–/–* mice		Enhanced ethanol conditioned place preference	(113)
		β-caryophyllene		Decreased ethanol consumption and preference	(114)
	Chronic mild stress	JWH015	20 mg/kg	Enhanced alcohol intake	(74, 115)
	Alcohol consumption			Increased amygdala expression of CB2Rs	(116)
Autism Spectrum Disorder	BTBR T + tF/J			Increased level of CB2AR mRNA	(38)

**TABLE 2:** Clinical data on the role of CB2Rs in neuropsychiatric disorders.

Disorder	Study	Outcome	References
Anxiety	Children with anxiety	Significant relationship between rs2070956 polymorphism and treatment outcome	(83)
Depression	Japanese depressive patients	High incidence of Q63R polymorphism of CB2R	(37)
	Postmortem study	Reduced expression of CB2R gene	(89, 90)
Schizophrenia	Japanese schizophrenia patients	Significant association between *CNR2* polymorphisms rs12744386 and rs2501432	(98)
Addiction	Japanese alcoholic patients	Single nucleotide polymorphism R63Q in *CNR2* gene	(74)
Autism spectrum disorder	Children with autism spectrum disorder	Upregulation of expression of *CNR2* gene in peripheral blood mononuclear cells	(121)
Eating disorders	Patients with eating disorders	No change in CB2R mRNA level in blood of the subjects	(135, 136)
	Japanese patients with eating disorder	Link between *CNR2* gene polymorphism and eating disorder	(137)

**TABLE 3:** Pre-clinical data on the role of CB2Rs in neurologic and neurodegenerative disorders.

Disorder	Model/paradigm	CB2R manipulation	Dose used	Outcome	References
Alzheimer’s disease	AβPP/PS1 transgenic mice	JWH133	0.2 mg/kg	Reduced tau hyperphosphorylation, induced vasodilation	(148, 149)
		JWH133	0.2 mg/kg	Enhanced brain glucose uptake	(150)
		*N*-linoleyltyrosine	15, 30, and 60 mg/kg	Protected neurons against Aβ injury	(151)
	APPSw/Ind	JWH133	0.2 mg/kg	CB2R activation blocks NMDA signaling in activated microglia	(152)
	APP/PS1 mice	JWH015	0.5 mg/kg	Improved novel object recognition, regulation in microglia-mediated neuroinflammation and dendritic complexity	(153)
	Double transgenic APP/PS1 mice	1-((3-benzyl-3-methyl-2,3-dihydro-1-benzofuran-6-yl) carbonyl) piperidine (MDA7)	15 mg/kg	Inhibited microglia activation, enhanced clearance of Aβ and decrease level of CB2R expression	(154, 155)
Parkinson’s disease	MPTP model	AM1241	0.75–12 mg/kg	Prevented neurodegeneration	(165)
	Lipopolysaccharide (LPS) model	*Cnr2–/–*		Enhanced activation of microglia	(170)
	6-hydroxydopamine (6-OHDA)	Δ^9^-THCV	2 mg/kg	Reduced motor inhibition and loss of TH-positive neurons caused by 6-OHDA, reduced CB2Rs up-regulation	(171)
	Lipopolysaccharide (LPS) model			Exhibited a greater up-regulation of CB2Rs	
	MPTP model	JWH015	4 mg/kg	Protected MPTP induced neurodegeneration and suppress microglia activation	(172)
	Rotenone (ROT) animal model	β-caryophyllene	50 mg/kg	Reduced oxidative stress and neuroinflammation	(173)
Huntington’s disease	Transgenic R6/2 mouse model			Elevated CB2R expression in the hippocampus, striatum and cerebellum	(178)
	Malonate rat model	*Cnr2–/–*		CB2R activation protected striatal neuron degeneration	(179)
	R6/2 mice	*Cnr2–/–*		CB2R agonist suppresses motor deficits, synapse loss, and CNS inflammation	(180)
	R6/2 mice	*Cnr2–/–*		Increased CB2R expression in striatal microglia	(181)
Multiple Sclerosis	Experimental autoimmune encephalomyelitis (EAE)			Enhanced CB2R expression	(184)
		HU308	15 mg/kg	Reduced symptoms, axonal loss and microglia activation	(185)
		O-1966	1 mg/kg	Reduced immune cell invasion and improved neurologic functions	(186)
		β-caryophyllene	25 and 50 mg/kg	Inhibited activation of immune cells and diminished axonal demyelination	(187)
	Theiler’s murine encephalomyelitis virus model	JWH015	0.6, 0.9, and 1.2 mg/kg	Improved the neurological deficits in a long-lasting way, induced anti-inflammatory response	(189, 190)
Amyotrophic lateral sclerosis	TDP-43 transgenic mice			Upregulation of CB2Rs	(197)
	G93A-SOD1 mice	AM1241	0.1 and 1 mg/ml	Slowed motor neuron degeneration and preserved motor function	(194)
			1 mg/kg	Delayed disease progression	(195)
Epilepsy	Palmitoylethanolamide (PEA)	AM630	2.5–40 μg/kg	Blocked palmitoylethanolamide induced seizure	(204)
	Pilocarpine, pentylenetetrazole and isoniazid-induced epileptic seizure models	β-caryophyllene	200 mg/kg	Improved seizure activity	(205)
	Pentylenetetrazole (PTZ) or methyl-6,7-dimethoxy-4-ethyl-beta-carboline-3-carboxylate (DMCM)	HU308	0.2–1 mg/kg	No antiseizure effect	(206)
		AM630	0.2–1 mg/kg	Didn’t increase seizure activity	
	Pentylenetetrazole (PTZ)	AM1241	1 and 10 μg/μl	Increased seizure intensity	(207)
	Pentylenetetrazole (PTZ)	*Cnr2–/–*		Increased susceptibility to seizure	(208)
		JWH133	3 mg/kg	Didn’t alter seizure susceptibility	
Traumatic brain injury	Controlled cortical impact (CCI)	O-1966	1 mg/kg	Attenuated blood–brain barrier disruption and neural degeneration	(210)
			1 mg/kg	Induced acute neuroprotection	(211)
	Experimental closed-head injury (CHI)	HU-910	0.1–10 mg/kg	Enhanced neuroprotection and neurobehavioral recovery	(212)
	Experimental closed-head injury (CHI)			Increased expression of CB2Rs	(62)
	Controlled cortical impact (CCI)	JWH133	1.5 mg/kg	Reduced white matter injury	(213)

**TABLE 4:** Clinical data on the role of CB2Rs in neurologic and neurodegenerative disorders.

Disorder	Study	Outcome	References
Parkinson’s disease	PD patients	Elevated CB2Rs in microglia cells in the substantia nigra	(174)
Multiple sclerosis	Human postmortem specimen	Enhanced microglia cells	(191)
	Postmortem brain tissue	Enhanced expression of CB2Rs	(192)
Amyotrophic lateral sclerosis	Postmortem specimen	Upregulation of CB2R	(191)

### Role of Cannabinoid Type 2 Receptors in Psychiatric and Emotionality Disorders

#### Anxiolysis and Anxiety-Related Disorders

Anxiety is a behavior associated with the anticipation of possible threats that provides coping mechanism to prevent stressful or harmful situations (77). There is increasing preclinical evidence for the involvement of the ECS in controlling mood and emotional behavior, with anecdotal evidence from cannabis users with different subjective effects. The ECS has a pivotal role in the regulation of mood disorders (78) and CB2Rs are expressed in the amygdala, hippocampus, prefrontal cortex and hypothalamus. These brain areas are involved in regulating anxiety like behavior and this suggests the potential role of CB2Rs in regulation of emotional responses (13, 32, 35, 79). Using the a two-compartment black and white chamber- and elevated plus maze test for assessing anxiety-like behavioral responses, we have reported on the effect of CB2R ligands on stress-induced anxiety-related behavior in mice. Our result showed that acute administration of JWH-015 (1–20 mg/kg) which has more affinity for binding to CB2Rs than to CB1Rs dose dependently induced an anxiogenic response in the black and white box but attenuated stress-induced gender-specific aversion to the open arms of the elevated plus maze (34, 35). Using rodent models of acute and chronic anxiety-like behavioral responses, Valenzano et al. (80) demonstrated that administration of GW405833 (100 mg/kg), a CB2R agonist, induced anxiolytic-like effects in the marble burying test that was not reversed by the administration of CB2R antagonist. However, in a mouse stress model, chronic mild stress (CMS) increased anxiety-like behavior responses in Zero maze test paradigm, and treatment with JWH-015 (20 mg/kg) reduced the anxiety-like behavioral effects similar to the effects of the antidepressant, fluvoxamine (81).

The involvement of CB2Rs in anxiety-like behavior was described by studies using selective CB2R antagonists. Acute administration of AM630 (1, 2, or 3 mg/kg) in mice induced anxiogenic-like behavioral effects, whereas with chronic administration anxiolytic-like effects were reported using the light-dark box and the elevated plus maze test (56). Treatment with AM630 (3 mg/kg) worsened the anxiety-like behavioral responses induced by CMS, contrary to the effects observed with JWH-015 treatment as described above (82). Interestingly, studies using transgenic mice overexpressing CB2R in the CNS (CB2xP mice) showed lack of effect against anxiogenic-like stimuli in the light-dark box and the elevated plus-maze behavioral tests (56). In agreement with this, another study using mice lacking the CB2R gene (*Cnr2*–/– mice) revealed that CB2R gene knock out mice developed higher levels of anxiety-like behavioral responses in both tests (59). We have investigated the involvement of CB2Rs in emotionality test using dopamine neuron cell-type specific CB2R conditional knockout mice (DAT-*Cnr2* cKO). The study utilized the elevated plus-maze and the two compartment black/white box behavioral tests (14) and the results demonstrated that the DAT-*Cnr2* cKO mice experience significant anxiety-like behavior compared to the wild type mice.

While the mechanism and the involvement of the ECS in mood and emotionality requires more investigation, clinical studies on the role of CB2Rs on anxiety-like behavior are very scarce. But, one study done on children with a primary anxiety disorder investigated whether genetic variation in the ECS explained individual differences in response to cognitive behavioral therapy (83). The study revealed that there is a relationship between the rs2070956 and rs2501431 polymorphisms of *CNR2* gene and a reduced treatment outcome in children with anxiety disorders. While rs2070956 and rs2501431 polymorphisms are functionally unknown, however, rs2501431 is located in the main exon of CNR2 gene. The results obtained from animal and clinical studies show a role of CB2Rs in modulating anxiety-like behavior, however, there is a need for further studies in the determination of the therapeutic potential of CB2Rs in anxiety and mood disorders.

#### Endocannabinoidome-Cannabinoid Type 2 Receptors and Depression

A new approach and targeting the eCBome and psychopathology is increasing not only in basic but also in clinical trials and research with increasing use of cannabis and cannabinoid analogs in children and adult population. There are different types of depression with co-morbidities with mental and neurological disorders. Major depression is characterized by depressed mood and anhedonia – a lack of pleasure, including other symptoms like reduced appetite and libido and disturbed sleep. Preclinical and clinical studies showed that the ECS is responsible in modulating depression with reports that there is a negative correlation between the ECS signaling and depression (84). In animal models of depression and depressed patients, changes have been observed in eCB levels. The withdrawal of rimonabant, a CB1R antagonist, used for the treatment of obesity due to risk of suicide and depression has received increased interest in the role of the CB1R in affecting mood and affective behavior. Some studies have investigated the role of CB1Rs in depression with more basic and clinical components, and factors like stress, interaction with the endocrine system and the use of cannabis and depression with revealing outcomes. Whereas, the involvement of the CB2R in affective disturbances has not received much attention (52). Previous studies indicate that overexpression of CB2R provoked depression-like response. CB2xP mice (overexpressing CB2R) reduced immobility time in acute models of depression (tail suspension and novelty-identification and feeding tests) (80). On the contrary, the results found with the dopamine neuron cell-type specific CB2R conditional knockout (DAT-*Cnr2*) (14) and *Cnr2*–/– mutant (59) mice demonstrated depressive-like behavior indicated by enhanced immobility time in the tail suspension and forced swimming tests. This discrepancy might be due to individual and species differences between the strains used and/or differences in the animal model utilized. A pharmacologic study using GW405833, a CB2R agonist, demonstrated that acute administration of the agonist did not alter time spent immobile in the forced swim test (85).

It is important to point out the validity and the mixed and conflicting responses in the use of some of these animal models of depression that have been questioned in the scientific community. Nevertheless, the CMS is commonly used animal model of depression with similar behavioral and physiological effects observed in clinical settings in patients with depressive disorders (86–88). The involvement of CB2R in depression was demonstrated in a study using mice subjected CMS. The results showed that CB2R protein levels measured by immunoblotting in whole brain extract were enhanced in mice subjected to CMS for a period of 4 weeks (36, 45). However, another study showed a reduction in the level of CB2R mRNA in the hippocampus of mice subjected to CMS for 7–8 weeks, compared with non-stressed controls (80). The contradiction might be due to differences in the time of exposure to CMS and variations in the brain regions used to measure the level of CB2R mRNA. The CMS protocol is characterized by reduced intake of sucrose, used as a measure of anhedonia-like response, a hallmark of depression. Studies using transgenic mice that over-express the CB2R showed that there is no change in sucrose consumption and immobility time in mice subjected to tail suspension test, respectively (80). But in another study done to evaluate the effect of CB2R ligands on sucrose consumption, injection of JWH-015, a CB2R agonist, daily or AM630, a CB2R antagonist, did not alter CMS-induced decreases in sucrose consumption (36, 37). Taken together, results from the preclinical studies indicate the possible behavioral and molecular role played by CB2Rs in CMS.

There is limited literature on the role of CB2Rs in depression in humans. In one study done on Japanese patients with depression, the authors found that there is a high incidence of the Q63R polymorphism of CB2R gene, this polymorphism could alter the receptor function and reduces the physiological responsiveness to CB2R ligands (37). Furthermore, a reduction in the expression of CB2R gene was documented in brain regions involved in regulating emotionality like the amygdala and the prefrontal cortex in a postmortem study performed in suicide patients (89) and in patients with major depression (90), supporting a role that CB2Rs could play in depression and the potential of these receptors as a new target for the treatment of depressive disorders, but requires additional modeling and clinical investigations.

#### Endocannabinoidome-Cannabinoid Type 2 Receptors in Psychosis

There are risks for harm with rising cannabis and cannabinoid medical use, and the widespread availability for recreational consumption creates the potential for psychotic disorders especially in adolescents whose brains are still developing and in those with psychosis. Thus, early exposure can trigger onset of psychosis in such vulnerable adolescents. Schizophrenia a type of psychotic disorder that is characterized by thinking disturbances, hallucinations and delusions is associated with genetic and epigenetic risk factors. Disturbances in the regulation of the ECS with altered levels of eCBs was associated with the development of schizophrenia. Indeed densities of eCB receptors and levels of eCBs have been suggested as possible biomarkers in neuropsychiatric disorders (83). Studies also revealed that the causal disturbances of neural processes in adulthood by early cannabinoid exposure are linked to psychiatric risk like schizophrenia (91). Therefore, the role of cannabinoid self-medication hypothesis, early exposure to cannabis and onset of psychosis in vulnerable adolescents has fueled research interests in changes in the ECS in neuropsychiatric disorders. Recently there is also an increase interest in the use of herbal, synthetic and eCBs for symptomatic management of schizophrenia (92). Studies using animal models of schizophrenia implicated the involvement of CB2R in schizophrenia. Early maternal deprivation in rodents is a model for neurodevelopmental stress and there are some reports indicating that maternal deprivation affects the ECS and that these changes may account for the proposed schizophrenia-like phenotype (93, 94). A study to evaluate the expression of CB1R and CB2R in the hippocampus showed that maternal deprivation induced a significant increase in CB2R immunoreactivity in the hippocampal areas, indicating possible involvement CB2Rs in neurodevelopmental mental illnesses such as schizophrenia (95).

The pre-pulse inhibition (PPI) test is one of the tests used to assess attention deficits associated with schizophrenia (96). Methamphetamine or MK-801, a non-competitive *N*-methyl-D-aspartate (NMDA) glutamatergic receptor antagonist, are two widely used animal models of schizophrenia that mimic effects observed in schizophrenic individuals (97). Studies showed that AM630, a CB2R antagonist, did not affect PPI on its own, but it did enhance the MK-801- and methamphetamine -induced decrease in PPI and increase in locomotor activity (98). Another study found that administration of JWH015 in doses of 1, 3, and 10 mg/kg enhanced PPI impairment caused by MK-801 (99). CB2R’s role in schizophrenia was investigated in genetically engineered mice. It was reported that *Cnr2*–/– mice were more susceptible to hyperlocomotion caused by acute cocaine exposure, as well as PPI and cognitive changes. Furthermore, in these mutant *Cnr2*–/– mice, treatment with the antipsychotic risperidone normalizes PPI readings (59, 100). Interestingly another study showed that CB2R is required for the antipsychotic effects of acetylcholine muscarinic 4 (M4) receptor agonists like UV0467154 (101). This study found that stimulating M4 receptors increased the release of eCBs while inhibiting dopamine release, implying that M4 agonists have a role in antipsychotic efficacy. As a result, inhibiting CB2Rs with the antagonist AM630 would prevent the antipsychotic effects of M4 agonists. Clinical studies have also shown that CB2R is involved in schizophrenia. In Japanese schizophrenia patients, the *CNR2* polymorphisms rs12744386 and rs2501432 were found to be significant (98) and the result also showed that both risk alleles of the two polymorphisms associated with schizophrenia were related to the direction of lower functioning of *CNR2* and poor responses to CB2R ligands. As there is, yet no major evidence supporting the use of cannabinoids targeting other components of the eCBome, targeting the CB2R may play a critical role in modulating psychotic symptoms. In schizophrenia and some addictive disorders discussed below CB2Rs was associated with comorbidity of schizophrenia and cannabis dependence with polymorphisms in *CNR2* gene implicated as a risk factor. Therefore, controlled clinical trials are need to determine the role of CB2Rs in the treatment or as an adjunctive strategy in schizophrenia.

#### Cannabinoid Type 2 Receptors in Addiction

The efficacy and success of cannabinoid pharmacotherapy in drug addiction is at best with mixed results. Some reports show that cannabis use may be effective in curbing alcohol and opioid addictions (102). Paradoxically, with the increased prevalence of cannabis use globally, and self-medication hypothesis, associations between cannabis use, cannabis use disorder (CUD) and mood disturbances have increased (103). Cannabis use has been linked to psychiatric disorders including manic, psychotic and symptoms of depression, but the underlying mechanism remains unclear, with no currently approved treatment of CUD. Evidence from a number of preclinical and clinical studies show that the ECS is recognized as a neural substrate in the effects of classical drugs of abuse associated with drug addiction in vulnerable individuals. The discovery of this previously unknown but ubiquitous ECS, and that CBRs are encoded in the human genome on chromosome 1 and 6 has led to the changing landscape on cannabinoid research and providing scientific evidence on the potential therapeutic benefits targeting the ECS. However, studies also showed the involvement of the ECS in addictive behavior (104, 105) and the association of early childhood and adolescent exposure to cannabinoids with addictive behavior (91, 106). There are still gaps in our understanding, but the discovery of the expression of CB2Rs in brain regions involved in drug addiction, such as the ventral tegmental area (VTA), nucleus accumbens (NAc), amygdala and hippocampus unraveled and encouraged investigation of the role of CB2Rs and their potential therapeutic target in drug addiction (107, 108). Pharmacologic and genetic studies showed that CB2Rs are involved in the effects of psychostimulants like cocaine, amphetamine and methamphetamine. CB2R is involved in cocaine motor sensitization in studies employing genetically modified mice. Mice overexpressing CB2R in the (CB2xP) showed reduced hyperlocomotor effects in response to acute cocaine administration and were less susceptible to the motor sensitization after repeated cocaine administration (109). Interestingly, mice lacking the CB2R (Cnr2–/–), which have increased sensitivity to cocaine-induced hyperlocomotion, had the opposite effect (59).

A study aimed to evaluate the effects of psychostimulants in dopamine neuron CB2R conditional knockout (DAT-*Cnr2* cKO) mice on locomotor activity showed that mice had increased cocaine, amphetamine and methamphetamine-induced hyperactivity, without psychostimulant-induced sensitization when compared to wild type controls. In addition, the authors reported that cocaine, amphetamine, and methamphetamine produced robust conditioned place preference (CPP) in both DAT-*Cnr2* cKO and wild type mice (58). In stark contrast, studies using JWH133 revealed systemic and local administration of JWH133 into the NAc blocked cocaine hyperlocomotion in mice (33, 110). In line with this, a study revealed that JWH133 inhibited cocaine and nicotine-induced CPP in wild type mice (58). However, in another investigation AM630 was found to have no effect on the locomotor effects after an acute or repeated cocaine administration in rats (110, 111). These inconsistencies could be explained by differences in animal species and the methods utilized. Since cocaine produced place aversion rather than place preference in CB2xP animals, and these transgenic mice showed an impairment in the acquisition of cocaine self-administration (109), CB2Rs appear to be implicated in the reinforcing features of cocaine. Evidence also showed that intranasally or intra-accumbens administration of JWH133 prevented intravenous cocaine self-administration (33). The involvement of CB2Rs in a model of addictive behavior has been demonstrated by the study using the natural CB2R agonist β-caryophyllene (BCP). The results showed that BCP attenuated methamphetamine self-administration in rats which was partially blocked by AM630 (112). The study also revealed that deletion of CB2Rs blocked low dose BCP-induced reduction in methamphetamine self-administration.

There are also investigations on the involvement of CB2Rs in the modulation of alcohol consumption and reward. The injection of BCP lowered ethanol CPP and ethanol intake in mice, as well as increased voluntary ethanol consumption in the two bottle paradigm and incentive to drink in the oral ethanol self-administration in the mutant *Cnr2*–/– mice (113, 114). In addition, sub-chronic injection of JWH015 enhanced alcohol intake in mice that had previously been exposed to chronic stress, with no impact in mice that had not been exposed to chronic stress (74, 115). A recent study also demonstrated that alcohol exposure during adolescence induced increases in the amygdala expression of the CB2Rs in rats (116).

A substantial incidence of the single nucleotide polymorphism R63Q in the *CNR2* gene locus was observed in a cohort of Japanese alcoholic patients in clinical investigations (74). This single nucleotide polymorphism causes a missense mutation in the first intracellular domain and causes a structural change, which could alter the receptor function and reduces the physiological responsiveness to CB2R ligands (107). More recently, Navarrete et al. extensively complied significant appraisal of CB2R involvement in the treatment of substance use disorders (SUDs), including alcohol, psychostimulants (cocaine and nicotine) with emphasis on the CB2R species differences in the effects of the SUDs covered (117). They concluded that the development of pharmacotherapeutic agents targeting the CB2Rs might offer novel approaches when combined with existing options (118). All the above data support a role of CB2Rs in modulating the addictive effects of alcohol and different psychostimulants, indicating that CB2Rs might be targeted in the treatment of drug addiction. Therefore, there is now increased focus and interest in CB2Rs in drug and alcohol addiction with increasing reports indicating their functional expression not only in reward circuits but also on the CB2R-neuro-immune crosstalk and signaling in neuropsychiatric disorders. With global increase in medicinal and recreational cannabis use, there is potential for increased CUD and therapeutic challenge as the frequency of cannabis use is a risk factor for developing and predicting CUD. Of note, no direct clinical studies have focused on targeting CB2Rs in CUDs.

### Role of Cannabinoid Type 2 Receptors in Autism Spectrum Disorder

Autism spectrum disorder (ASD) has early childhood onset with a lifelong progression. It is characterized by poor social skills, deficits in communication as well as by stereotypic behaviors, (119). The ECS plays a significant role in ASD. A review on the role of the ECS in autism found documentation that endocannabinoid signaling plays a key role in many human health and disease conditions of the CNS (120). However, there is limited literature, pre-clinical and clinical, on the role of CB2Rs in ASD. CB2Rs were also identified as a possible target for autism in preliminary research. Indeed, in the cerebellum of BTBR T + tF/J, a mouse model of ASD, there was an increased level CB2A mRNA expression, but not the CB2BR, gene-transcript isoforms (38).

A controlled study of ASD done on autistic children showed that gene expression for *CNR2* but not *CNR1* was up-regulated in peripheral blood mononuclear cells (PBMCs). The result indicated that *CNR2* gene expression was significantly higher in individuals with ASD compared to controls. In addition, the gene expression for one of the enzymes responsible for synthesis of the endocannabinoid anandamide (AEA) (NAPE-PLD) was significantly lower in individuals with ASD. This could have caused an increase in CB2Rs resulting from a decrease in AEA synthesis which may be indicative of decreased ECS tone in ASD (121). While there a multifactorial and putative link of the ECS to ASDs, there are no solid biomarkers, and our investigations using BTBR T + tF/J, and other preclinical mouse models rely on characterization of behavioral alterations (38). So there is a need of a thorough behavioral characterization of mouse models of ASD to demonstrate the therapeutic effects of pharmacological treatments targeting the ECS (122). It is important that more basic and clinical research and trials may unravel etiology of ASDs that involve multigenetic risk factors and environmental neurodevelopment factors *in utero* including use of drugs, exposure to chemicals, infections, diet and stress (123–125). Further basic and clinical research has also to be done on the role of CB2Rs in ASDs before claiming this receptor as a potential therapeutic target in ASDs.

#### Eating Disorders

The ECS modulates appetite – a basis for adjunctive cannabinoids use to counter AIDs cachexia and regulates the metabolism of glucose in the pancreas and liver (126–128). It involved in the gaging appetite value, control and associated with weight and obesity supporting data on the modulation cannabinoids in food intake. Food craving often referred to as munchies after smoking or ingesting cannabis has implicated the ECS in food intake. A striking evidence was the approval and use of the CB1R antagonist, acomplia as an antiobesity medication in Europe and was withdrawn because of suicide, and other limiting psychiatric side effects. Now with significant technological advances and progress in eCBome research, it may be feasible to produce ligands with opposing activation profiles of CB1R and CB2R with high degree of selectivity in obesity, neuropsychiatric and neurodegenerative disorders. ‘Diabesity’ refers to the relationship between diabetes and obesity (129, 130) that is in part linked with ECS signaling in adiposity and metabolism. This indicates that dysregulation of the gut ECS and altered levels of eCBs is associated with obesity and metabolic syndrome. The physiological roles of the ECS in the gastrointestinal tract (GIT), adipogenesis and lipogenesis provides an increasing link in dysfunction of the ECS with obesity, metabolic syndrome and other disturbances in the periphery (131, 132). The most common eating disorders are anorexia nervosa (AN) and bulimia nervosa (BN) presents abnormal eating behaviors that generally result in severe food restriction with episodes of binge eating and vomiting without significant changes of body weight in BN and a dramatic loss of body weight in AN (119). The hypothalamic eCBs have been shown to modulate eating behavior in animals and humans, thus indicating the role of the ECS in the pathophysiology of eating disorders (133, 134). A study done on 20 women with AN, 23 women with BN, and 26 healthy women, the levels of CB1R and CB2R expression were examined. The study found no differences in CB2R mRNA levels in the blood of AN and BN patients when compared to controls, with no significant difference between the two groups (135, 136). So far, there is only one study done on human genetic association to identify whether the *CNR2* gene is involved in eating disorders (137). 204 individuals with eating disorders (94 AN and 111 BN) and 1876 healthy Japanese volunteers participated in the study. A non-synonymous *CNR2* polymorphism, which reduces the physiological responsiveness to CB2R ligands, was found to be linked to both AN and BN. The R allele is substantially more prevalent in people with eating disorders than in controls, according to the findings. Furthermore, there was no change in allele frequency between patients with AN and BN when they were separated. The study demonstrates a link between CB2Rs and eating disorders. There is a paucity of literature on the role of CB2Rs in eating disorders, so there is a need for more research to corroborate these findings.

### Role of Cannabinoid Type 2 Receptors in Neurologic and Neurodegenerative Disorders

#### Alzheimer’s Disease

Alzheimer’s disease (AD) is characterized by abnormal accumulation of β-amyloid (Aβ) in senile plaques in the brain that causes cognitive impairment, memory loss, and behavioral changes due to neurodegeneration and inflammation (138). Studies demonstrate that the ECS is involved in the regulation of memory (139, 140) and ECBs have been shown to decrease Aβ-induced microglia activation and neuroinflammation (141–143). Recently the CB2Rs have attracted attention in AD investigation because of its expression in immune cells and its enhanced expression during inflammation. Studies showed that there is increased expression of CB2Rs in brain tissue in AD patients and mouse models expressing pathogenic variants of amyloid precursor protein (APP) (141, 144, 145). Previous research found that genetic deletion of CB2Rs increased Iba1 staining and exacerbated soluble A42 and plaque deposition (146), implying that CB2Rs play a key role in preventing amyloid plaque pathology in AD (147). In mice, administration of JWH-133 improved cognitive impairment, inhibited neuroinflammation and oxidative stress, decreased tau hyperphosphorylation, induced vasodilation (148, 149), and enhanced glucose uptake, indicating that CB2R agonists could be used as nootropics (150). A recent study assessing the role of AEA analog-*N*-linoleyltyrosine (NITyr) in APP/PS1 mice mimicking the AD model showed that NITyr protected neurons against Aβ injury which is mainly mediated by the CB2Rs (151). Interestingly Rivas-Santisteban et al. (152) demonstrated CB2R activation blunted NMDA receptor-mediated signaling in primary hippocampal neurons from APP_Sw/Ind_ mice model of AD, suggesting a role of CB2Rs in AD pathogenesis. In addition, in AD model mice, CB2R activation by JWH-015 was found to improve new object recognition abilities while also regulating microglia-mediated neuroinflammation and dendritic complexity in a region-specific manner (153). Parenteral administration of 1-((3-benzyl-3-methyl-2,3-dihydro-1-benzofuran-6-yl) carbonyl) piperidine (MDA7), a novel selective CB2R agonist, inhibited the activation of microglial cells and astrocytes, decreased the level of expression of CB2R, enhanced the clearance of Aβ, and improved cognition and memory in rodent AD models (154, 155).

In various *in vitro* and *in vivo* AD models, CB2R activation reduced levels of neurotoxic factors and pro- inflammatory mediators produced by reactive astrocytes and microglial cells, stimulated microglial proliferation and migration, and decreased Aβ levels (156–158). Despite a large body of evidence for the involvement of CB2Rs in reducing and processing Aβ in a mouse model of AD, it is difficult to determine the therapeutic value of cannabis-based medicines in AD (159). This might be because of lack sufficient randomized clinical trials and inconsistent and inconclusive evidence. There are also reports that showed that mice with CB2Rs deficiency showed a reduction in microglial cells and macrophages, reduced expression levels of brain pro-inflammatory cytokines, diminished concentrations of soluble Aβ40/42, improved cognitive and learning deficits (141). In few clinical studies, there are contradictory reports on the potential of CB2R ligand in AD and further research is required to determine the potential role of CB2Rs in humans and in animal models of AD.

#### Parkinson’s Disease

Parkinson’s disease (PD) is a movement disorder characterized by degeneration of dopaminergic neurons that results in motor dysfunction (160, 161). Disease progression is characterized by inflammation through activation of microglia (162) and an increase in cytokines (163, 164). Accumulated evidence suggests that there is a strong potential for the ECS that could provide neuroprotection against acute or chronic neurodegenerative disorders (165–169). CB2R levels were significantly elevated in animal models of PD and postmortem studies of PD patients and this increase correlated significantly with an increase in microglial activation, indicating the possible role of CB2Rs in PD (64, 170). On the other hand, studies showed downregulation of CB2Rs in the substantia nigra and hippocampus 3 weeks after 1-methyl-4-phenyl-1,2,3,6-tetrahydropyridine (MPTP) injection in mice with MPTP-induced parkinsonian syndrome. This discrepancy might be due to differences in the animal model used, duration of treatment and/or brain regions studied. In addition, AM1241, the selective CB2R agonist, has been shown to regenerate DA neurons after the neurotoxic effect of MPTP treatment (165). Furthermore, mice lacking the CB2R showed enhanced activation of microglial cells and much more intense deterioration of tyrosine hydroxylase (TH)-containing nigral neurons in animal models of PD (170), which supported the potential neuroprotective role of CB2Rs.

In animal models of PD, Δ^9^-THCV, a CB2R agonist, reduced motor inhibition caused by 6-hydroxydopamine (6-OHDA) and the loss of TH–positive neurons caused by 6-OHDA lesion in the substantia nigra after an acute and chronic administrations, respectively. However, CB2Rs were poorly up-regulated in the rat substantia nigra in response to 6-OHDA. By contrast, the substantia nigra of mice that had been injected with lipopolysaccharide (LPS) exhibited a greater up-regulation of CB2Rs. The authors suggest that Δ^9^-THCV caused preservation of TH–positive neurons probably through the involvement of CB2Rs (171).

In another study the protective effect of JWH-015 against MPTP-induced nigrostriatal degeneration and suppression of microglial activation/infiltration through activation of CB2Rs (172) was implicated. In addition, there are reports showing that BCP reduced oxidative stress and neuroinflammation, blocked gliosis and pro-inflammatory cytokine release, and lowered nigrostriatal degeneration in rotenone (ROT) induced animal model of PD produced (173). Like the pre-clinical studies, clinical studies revealed that the expression of CB2Rs in different brain cells is upregulated in PD patients. One study demonstrated that CB2Rs are elevated in microglial cells recruited and activated at lesioned sites in the substantia nigra of PD patients compared to control subjects (170). In another study the authors observed that CB2R was located in TH-containing neurons in the substantia nigra at levels significantly lower in PD patients than in controls (174). Thus, CB2Rs may be a promising pharmaceutical target for alleviating parkinsonian symptoms and slowing disease development in Parkinson’s disease.

#### Huntington’s Disease

Huntington’s disease (HD) is a neurodegenerative disease caused by the expansion of CAG triplet repeat (cytosine-adenine-guanine), in the gene encoding the protein huntingtin (*Htt*), which leads to cognitive decline and abnormal motor movements (chorea) (24, 175). Currently there is no effective treatment for HD and the need for new therapeutic targets is increasing (24). Because the ECS is abundant in the basal ganglia, activation or inhibition of the ECS signaling pathway may have a major impact on motor responses (176), and CB2R is emerging as a new therapeutic target for the treatment and early diagnosis of HD (177). In the transgenic R6/2, CAG repeat length Huntington chorea mouse model, CB2R expression was shown to be elevated in the hippocampus, brain, striatum, and cerebellum (178). Studies also found that mice lacking the CB2Rs were more sensitive to malonate than the control group. In addition, CB2R-defficient mice exhibited an enhanced onset of motor deficits and increased severity (179, 180). There is expression of CB2R that was increased in striatal microglia in a transgenic mouse model of HD and in patients. In addition, the genetic ablation of CB2R exacerbated HD and the administration of CB2R-selective agonists reduced striatal neurodegeneration through microglial activation (181). Taken together, CB2Rs are neuroprotective and might be a potential target and compounds that selectively activate CB2R might be utilized as a potential therapeutic agent in the treatment of HD.

#### Multiple Sclerosis

Multiple sclerosis (MS) is an autoimmune disorder characterized by inflammation, neurodegeneration, and demyelination of neurons with no effective therapy (182) and studies showed the role of CB2Rs in inflammatory conditions associated with MS (177, 183). Microglial activation was linked to CB2R overexpression in an experimental autoimmune encephalomyelitis (EAE) animal model of MS (184). HU-308, the CB2R-selective agonist, also dramatically reduced EAE symptoms, axonal loss, and microglial activation when given orally (185). O-1966, a CB2R agonist, was also found to reduce immune cell invasion, diminish white cell rolling and adhesion to cerebral microvessels and improve neurologic function following an insult on EAE progression (186). Additional study done using EAE animal models demonstrated that BCP significantly inhibit microglial cells, CD4+ and CD8+ T lymphocytes, cytokines and diminished axonal demyelination and modulated Th1/Treg immune balance through the activation of CB2Rs (187). Maresz et al. (184) also investigated that CB2R expression by encephalitogenic T cells reduced EAE associated inflammation. Furthermore, during EAE, CB2R-deficient T cells in the CNS showed reduced apoptosis, increased proliferation, and increased production of inflammatory cytokines, resulting in severe clinical illness. The Theiler murine encephalomyelitis virus- induced demyelinating disease (TMEV- IDD), which mimics central neuronal demyelination that occurs in MS, is another animal model of MS (188). In TMEV- IDD mice, CB1R and CB2R agonists showed an enhanced clinical benefits through immunomodulatory and anti- inflammatory mechanisms such as activation of CD4^+^ T cells, increase in regulatory CD4^+^ T cells in the CNS along with alterations in the cytokine and chemokine milieu (189, 190), which is another evidence for the role of CB2Rs in MS. Postmortem and clinical studies also showed that there is involvement of CB2Rs in MS. Yiangou et al. (191) indicated a higher level of microglia cells in human postmortem spinal cord specimens. Evidenced revealed that T lymphocytes, astrocytes, and both perivascular and reactive microglial cells were also observed to express CB2Rs in postmortem brain tissues from MS donors (192). These findings suggest that CB2Rs play a neuroprotective function in MS pathology, and targeting CB2Rs could aid in the management of symptoms and neurologic issues in MS patients.

#### Amyotrophic Lateral Sclerosis

Amyotrophic lateral sclerosis (ALS) is a cortical, brain stem and spinal cord motor neuron degenerative disease. Deterioration of both upper and lower motor neurons is the main feature of disease progression (141, 193). The involvement of CB2Rs in ALS has been demonstrated by different studies. A reduction in motor neuron degeneration and preservation of motor function in ALS was observed after selective activation of CB2Rs in animals (194–196). TDP-43 transgenic mice and postmortem specimen studies showed that there is upregulation of CB2Rs in activated microglia cells (191, 197). Studies also showed the survival interval after ALS onset was increased by 56% after the administration of the CB2R agonist AM1241 initiated at symptom onset (194). In addition, Kim et al. (195) reported a reduction in signs of disease progression when AM1241 was administered after onset of signs in an ALS mouse model [hSOD1(G93A)] transgenic mice. All these evidences indicate that CB2R has a role in preventing the progression of disease ALS. Therefore, CB2Rs might be considered a promising target for therapeutic approaches in ALS.

#### Epilepsy

Epilepsy is a common neurological illness, affecting more than 70 million individuals worldwide. The currently available anti-epileptic drugs are unable to control epileptic seizures in majority of patients (198), hence there is a need for a new therapeutic targets for effective anti-epileptic drugs. There is evidence on the role of the ECS in epilepsy (199, 200) and studies showed that CBD was effective in the treatment of epileptic seizures in preclinical (117) and clinical studies (201, 202). The role of CB2Rs in epileptic seizures has been documented in other studies (71, 203). A study showed that the selective CB2R antagonist AM630 inhibited palmitoyl ethanolamide (PEA) induced increase in the latency of seizure initiation and reduced the duration of seizures in an acute pentylenetetrazol (PTZ) rat seizure model (204). In addition, the administration of BCP was found to improve seizure activity in a mouse model (205). Despite the evidences showing the role of CB2Rs in controlling seizure in animals, few studies didn’t find a possible association between activation of CB2Rs and control of seizure. In a study using a HU-308, a CB2R selective agonist, HU-308 did not show an antiseizure effect and AM630 did increase seizure severity (206). Moreover, CB2R agonist AM1241 increased seizure intensity in a PTZ model (207) and AM630 and SR144528, can increase seizure susceptibility (206). Studies using genetically manipulated mice revealed that CB2R knockout mice show enhanced epileptic susceptibility, and a reduction in CB2R activity was associated with increased susceptibility (208). Taken together, accumulated evidence support the involvement of CB2Rs in the pathophysiology of epileptic seizures and hence CB2Rs might serve as a possible target for the treatment of epilepsy.

#### Traumatic Brain Injury

Traumatic brain injury is caused by mechanical injury of the brain that results in formation of hematoma that ultimately leads to long-term complications and death (209). Evidence shows the involvement of CB2Rs in modulating the pathophysiology of TBI. Studies using the CB2R agonist, O-1966, in mice with TBI indicated that administration of O-1966 attenuated blood-brain barrier disruption, neuronal degeneration (210) and induced acute neuroprotective effects (211), supporting a role of CB2Rs in the management of TBI. There are also reports showing an increase expression of CB2Rs in mice with TBI associated with edema neurologic deficits. In that study the authors found a positive correlation between the expression of CB2Rs and TBI (62). In another study aimed at evaluating the potential effects of the CB2R agonists, HU-910 and HU-914 in the pathophysiology of TBI in mice, Magid et al. (212) demonstrated an enhanced neuroprotection and neurobehavioral recovery. In a recent study the selective CB2R agonist, JWH133, protected white matter injury via PERK signaling in a rat model of traumatic brain injury (213–218).

## Limitations and Future Perspectives

The growing research interests in eCBome, disturbances in its regulation, altered levels of eCBs, determination of the crystal structures of CB1R and CB2R pharmacology, discoveries of associated genetic polymorphisms provides targets for therapeutic intervention in neuropsychiatric and neurodegenerative disorders in the era of global deregulation of cannabis for medical and recreational use. Indeed densities of eCB receptors and levels of eCBs have been suggested as possible biomarkers in neuropsychiatric and neurodegenerative disorders. With the increasing evidence that CB2Rs are involved in brain, the functional role of CB2R neuro-immune axis in pathophysiological signaling in the development of psychiatric disorders warrants further investigation. It is also noteworthy that the determination of the crystal structures of CB1R and CB2R reveals a yin-yang relationship and functional profile of CB2R antagonism verse CB1R agonism for further innovative development of ligands with multiple therapeutic outcomes (2, 18). The relationship shows that some compounds when tested *in vitro* in the cAMP assay were potent agonists for the CB1R. Conversely, all of these ligands behaved as CB2R inverse agonists or neutral antagonists in the same functional test (17). However, the physiological implications of such opposing activation profiles between CB1R and CB2R should be studied further for their therapeutic potential. This highlights another area where CB1R and CB2R seems likely to work both independently and/or cooperatively that will benefit from critical medical application development. There are limitations and concerns regarding the use of CB2R medication for neurological disorders, as they are abundantly expressed in the periphery, and may have peripheral side effects, while they may be useful in CNS disorders associated with neuroinflammation. Further, research evaluating the numerous compounds in cannabis along with terpenes and flavonoids, will add to our understanding of this natural eCBome in neuropsychiatric and neurodegenerative disorders and contribute to novel biomarkers and therapeutic agents in health and disease.

## Conclusion

With more research targeting the eCBome-CB2Rs, the pharmacogenomic basis might be personalized to different disorders that may require combination cannabinoids with drugs affecting other neurobiological targets. We now know that CB2Rs are found in the periphery and the CNS, but the absence of intoxicating effects of these receptors significantly increased attention for the investigation of these receptors as therapeutic targets in neuropsychiatric and neurodegenerative disorders. Currently there are no cures, and only partially effective treatment are available for most of these disorders and therefore, more research is warranted for new therapeutic targets of emerging eCBome using novel strategies including artificial intelligence (AI) that might have better prediction and therapeutic outcomes with minimal adverse effects. The rapid and explosion of cannabinoid research may also transform the current landscape in using *in vitro* and *in vivo* technologies that will enhance our understanding of the potential role of CBRs in neuropsychiatric and neurodegenerative disorders. Despite the progress recently, the withdrawal of the FAAH inhibitor during clinical trial and anti-obesity CB1R antagonist due adverse effects engenders cautionary approaches in clinical trials. It is encouraging that clinical trial targeting CB2R, and approval by the Food and Drug Administration (FDA) of nabiximols for epileptic seizures is making targeting components of the CB2R – ECS a pharmacotherapeutic strategy. The findings included in this review showed that CB2Rs are highly expressed in neuropsychiatric and neurodegenerative disorders and selective CB2R ligands have promising effect in symptomatic management of these disorders. Additional studies are required to evaluate the involvement of CB2Rs in these disorders using the full range of tools that are available to study the CB2Rs and their selective ligands in animal models as well as in controlled clinical trials. It is important that such future studies include translational and clinical profiles and *in vivo* and *in vitro* models that express human CB2Rs. Furthermore, careful evaluation of the side effects associated with chronic treatment of CB2R ligands will provide further insight into the potential role of CB2Rs in regulating neurophysiological and behavioral function.

## Author Contributions

HI and EO conceived the focus of the review. BK searched the literature and wrote the first draft of the manuscript. HI and YH assisted in literature research and writing the manuscript. EO supervised and revised the manuscript. All authors reviewed the manuscript and approved the final version.

## Conflict of Interest

The authors declare that the research was conducted in the absence of any commercial or financial relationships that could be construed as a potential conflict of interest.

## Publisher’s Note

All claims expressed in this article are solely those of the authors and do not necessarily represent those of their affiliated organizations, or those of the publisher, the editors and the reviewers. Any product that may be evaluated in this article, or claim that may be made by its manufacturer, is not guaranteed or endorsed by the publisher.

## Abbreviations

6-OHDA: 6-hydroxydopamine
AC: adenylyl cyclase
AD: Alzheimer’s disease
AEA: anandamide
AI: artificial intelligence
ALS: amyotrophic lateral sclerosis
AN: anorexia nervosa
APP: amyloid precursor protein
ASD: autism spectrum disorder
A β: β -amyloid
BCP: β -caryophyllene
BN: bulimia nervosa
CB1R: cannabinoid type 1 receptor
CB2R: cannabinoid type 2 receptor
CMS: chronic mild stress
CNS: central nervous system
CPP: conditioned place preference
D1Rs: dopamine type 1 receptors
EAE: experimental autoimmune encephalomyelitis
eCBome: endocannabinoidome
eCBs: endocannabinoids
ECS: endocannabinoid system
ERK: ½ extracellular signal-regulated kinase
FDA: food and drug administration
GIT: gastrointestinal tract
GPCRs: G-protein coupled receptors
HD: Huntington’s disease
JNK: Jun N-terminal protein kinase
Kir: inwardly rectifying potassium currents
LPS: lipopolysaccharide
MAPK: mitogen-activated protein kinase
MDA7: 1-((3-benzyl-3-methyl-2,3-dihydro-1-benzofuran-6-yl) carbonyl) piperidine
MPTP: 1-methyl-4-phenyl-1,2,3,6-tetrahydropyridine
MS: multiple sclerosis
NAc: nucleus accumbens
NITyr: *N*-linoleyltyrosine
NMDA: *N*-methyl -D-aspartate
PD: Parkinson’s disease
PEA: palmitoyl ethanolamide
PI3K: phosphoinositide 3-kinase
PPI: pre-pulse inhibition
PTZ: pentylenetetrazol
ROT: rotenone
TBI: traumatic brain injury
TH: tyrosine hydroxylase
TMEV- IDD: Theiler murine encephalomyelitis virus- induced demyelinating disease
VTA: ventral tegmental area
Δ ^9^-THC: delta-9-tetrahydrocannabinol.
